# Supplementary material for: Exploring the determinants of synergetic development of social organizations participating in home-based elderly care service: An SEM method
Source: PLoS One. 2020 Dec 31;15(12):e0244880. doi: 10.1371/journal.pone.0244880 (PMC7775099; doi:10.1371/journal.pone.0244880)
Supplement: Attachment — Submitted filename: PONE-D-20-20452_reviewer1.pdf [file pone.0244880.s002.pdf]

PLOS ONE

Exploring the Determinants of Synergetic Development of Social Organizations Participating in Home-based Elderly Care Service: An SEM Method

--Manuscript Draft--

**Manuscript Number:**	PONE-D-20-20452
**Article Type:**	Research Article
**Full Title:**	Exploring the Determinants of Synergetic Development of Social Organizations Participating in Home-based Elderly Care Service: An SEM Method
**Short Title:**	Exploring Determinants of Synergetic Development of Social Organization Participating in HECS
**Corresponding Author:**	Jingfeng Yuan<br>Southeast University<br>Nanjing, CHINA
**Keywords:**	synergetic development; impact factors; social organizations; home-based elderly care service (HECS); structural equation model (SEM)
**Abstract:**	The current aging service industry has problems in satisfying the ever-increasing demand for the home-based elderly care service (HECS). Social organizations participating in HECS seems to be a promising way to address these problems but also raises new challenges, like uncoordinated cooperation among stakeholders, which leads to low management efficiency and low service quality. Synergetic development can be promising to enhance the participation of social organizations and to improve social welfare. After identifying five determinants of synergetic development, a conceptual model was constructed. Based on a survey conducted in Nanjing, China, the concept model was tested by a structural equation model (SEM). The results indicated that stakeholder engagement plays a critical role in synergetic development in HECS, resource allocation can only be improved by institutional climate, and supervision capacity cannot facilitate information sharing . Finally, managerial implications and some suggestions about future directions are concluded.
**Order of Authors:**	Qiuhu Shao
	Jingfeng Yuan
	Junwei Ma
	Hongxing Ding
	Wei Huang
**Additional Information:**	
**Question**	**Response**
**Financial Disclosure**<br><br>Enter a financial disclosure statement that describes the sources of funding for the work included in this submission. Review the submission guidelines for detailed requirements. View published research articles from *PLOS ONE* for specific examples.<br><br>This statement is required for submission and **will appear in the published article** if the submission is accepted. Please make sure it is accurate.	The research is supported by the National Natural Science Foundation of China (NO. 71671042; receiver: Jingfeng Yuan), National Social Science Foundation of China (NO. 19CGL065; receiver: Wei Huang). Both Yuan and Huang play a role in the study design, data collection and analysis, decision to publish, or preparation of the manuscript.

Powered by Editorial Manager® and ProduXion Manager® from Aries Systems Corporation

**Unfunded studies**<br>Enter: *The author(s) received no specific funding for this work.*<br><br>**Funded studies**<br>Enter a statement with the following details:<br>• Initials of the authors who received each award<br>• Grant numbers awarded to each author<br>• The full name of each funder<br>• URL of each funder website<br>• Did the sponsors or funders play any role in the study design, data collection and analysis, decision to publish, or preparation of the manuscript?<br>• **NO** - Include this sentence at the end of your statement: *The funders had no role in study design, data collection and analysis, decision to publish, or preparation of the manuscript.*<br>• **YES** - Specify the role(s) played.<br><br>* typeset	
**Competing Interests**<br><br>Use the instructions below to enter a competing interest statement for this submission. On behalf of all authors, disclose any competing interests that could be perceived to bias this work—acknowledging all financial support and any other relevant financial or non-financial competing interests.<br><br>This statement **will appear in the published article** if the submission is accepted. Please make sure it is accurate. View published research articles from *PLOS ONE* for specific examples.	The authors have declared that no competing interests exist.

**NO authors have competing interests**<br><br>Enter: *The authors have declared that no competing interests exist.*<br><br>**Authors with competing interests**<br><br>Enter competing interest details beginning with this statement:<br><br>*I have read the journal's policy and the authors of this manuscript have the following competing interests: [insert competing interests here]*<br><br>* typeset	
**Ethics Statement**<br><br>Enter an ethics statement for this submission. This statement is required if the study involved:<br><br>• Human participants<br>• Human specimens or tissue<br>• Vertebrate animals or cephalopods<br>• Vertebrate embryos or tissues<br>• Field research<br><br>Write "N/A" if the submission does not require an ethics statement.<br><br>General guidance is provided below. Consult the submission guidelines for detailed instructions. **Make sure that all information entered here is included in the Methods section of the manuscript.**	N/A

Powered by Editorial Manager® and ProduXion Manager® from Aries Systems Corporation

**Format for specific study types**<br><br>**Human Subject Research (involving human participants and/or tissue)**<br>• Give the name of the institutional review board or ethics committee that approved the study<br>• Include the approval number and/or a statement indicating approval of this research<br>• Indicate the form of consent obtained (written/oral) or the reason that consent was not obtained (e.g. the data were analyzed anonymously)<br><br>**Animal Research (involving vertebrate animals, embryos or tissues)**<br>• Provide the name of the Institutional Animal Care and Use Committee (IACUC) or other relevant ethics board that reviewed the study protocol, and indicate whether they approved this research or granted a formal waiver of ethical approval<br>• Include an approval number if one was obtained<br>• If the study involved *non-human primates*, add *additional details* about animal welfare and steps taken to ameliorate suffering<br>• If anesthesia, euthanasia, or any kind of animal sacrifice is part of the study, include briefly which substances and/or methods were applied<br><br>**Field Research**<br><br>Include the following details if this study involves the collection of plant, animal, or other materials from a natural setting:<br>• Field permit number<br>• Name of the institution or relevant body that granted permission	
**Data Availability**<br><br>Authors are required to make all data underlying the findings described fully available, without restriction, and from the time of publication. PLOS allows rare exceptions to address legal and ethical concerns. See the PLOS Data Policy and FAQ for detailed information.	Yes - all data are fully available without restriction

Powered by Editorial Manager® and ProduXion Manager® from Aries Systems Corporation

A Data Availability Statement describing where the data can be found is required at submission. Your answers to this question constitute the Data Availability Statement and **will be published in the article**, if accepted.<br><br>**Important:** Stating ‘data available on request from the author’ is not sufficient. If your data are only available upon request, select ‘No’ for the first question and explain your exceptional situation in the text box.<br><br>Do the authors confirm that all data underlying the findings described in their manuscript are fully available without restriction?	
**Describe where the data may be found in full sentences. If you are copying our sample text, replace any instances of XXX with the appropriate details.**<br><br>• If the data are **held or will be held in a public repository**, include URLs, accession numbers or DOIs. If this information will only be available after acceptance, indicate this by ticking the box below. For example: *All XXX files are available from the XXX database (accession number(s) XXX, XXX.).*<br>• If the data are all contained **within the manuscript and/or Supporting Information files**, enter the following: *All relevant data are within the manuscript and its Supporting Information files.*<br>• If neither of these applies but you are able to provide **details of access elsewhere**, with or without limitations, please do so. For example:<br><br>*Data cannot be shared publicly because of [XXX]. Data are available from the XXX Institutional Data Access / Ethics Committee (contact via XXX) for researchers who meet the criteria for access to confidential data.*<br><br>*The data underlying the results presented in the study are available from (include the name of the third party*	The data underlying the results presented in the study are available from (include the name of the third party and contact information or URL).

and contact information or URL).<br>• This text is appropriate if the data are owned by a third party and authors do not have permission to share the data.<br><br>* typeset	
Additional data availability information:	Tick here if the URLs/accession numbers/DOIs will be available only after acceptance of the manuscript for publication so that we can ensure their inclusion before publication.

Powered by Editorial Manager® and ProduXion Manager® from Aries Systems Corporation

Manuscript Click here to access/download;Manuscript;MANUSCRIPT.docx

Exploring the Determinants of Synergetic Development of Social Organizations Participating in Home-based Elderly Care Service: An SEM Method

Qiuhu Shao [1], Jingfeng Yuan [1,*], Junwei Ma [1], Hongxing Ding [1], and Wei Huang[2]

[1] Department of Construction and Real Estate, School of Civil Engineering, Southeast University, Nanjing 210096, P. R. China; shaoqiuhu@126.com (Q.S.); 220181196@seu.edu.cn (J.M.); hxding@seu.edu.cn (H.D.)

[2] School of Civil Engineering, Sanjiang University, Nanjing 210012, P. R. China; hwmm1122@163.com (W.H.)

* Correspondence: jingfeng-yuan@seu.edu.cn; Tel.: +86-13851783195

Abstract

The current aging service industry has problems in meeting the ever-increasing demand for the home-based elderly care service (HECS). Therefore, it is necessary to call upon the participation of social organizations. However, social organizations participating in HECS has induced uncoordinated cooperation among stakeholders, which leads to low management efficiency and low service quality. Synergetic development can be considered a promising way to enhance the participation of social organizations and to improve social welfare. Based on the literature review, five determinants of synergetic development were identified: institutional climate, supervision capacity, stakeholder engagement, information sharing, and resource allocation. Then a conceptual model was constructed. According to a holistic questionnaire survey which was conducted in Nanjing, China, the concept model was tested by a structural equation model (SEM). The results indicated that stakeholder engagement plays a critical role in synergetic development in HECS, resource allocation can only be improved by institutional climate, and supervision capacity cannot facilitate information sharing. Finally, managerial implications and some suggestions about future directions are concluded.

Keywords: synergetic development; impact factors; social organizations; home-based elderly care service (HECS); structural equation model (SEM)

Introduction

According to the National Bureau of Statistics of China, the number of people aged 60 and over in China has exceeded 253 million at the end of 2019, accounting for 18.1% of Chinese total population [1]. With the growing number of the elderly, the demand for aging care raises new challenges. Therefore, it is necessary to promote the extension of elderly services to communities and families [2]. Traditionally, the elderly tend to rely on family members for care services. However, with adavanced social economies and profound changes in family structure, traditional family care cannot well address current problems in China, which requires the participatant of the government and social organizations. For increasing demands of aging care services, aging care institutions have already undergone significant modifications in sizes, but fail to provide satisfying services due to high operation costs, expensive prices, and unclear industry standards [3].

Facing new demographic structures, the government induces communities and social organizations to participate in home-based elderly care service (HECS). Social organizations are integrate to social economies and act as important service providers in China. They could provide home-based care and emergency services, and promote overall quality of life outcomes for the elderly with advanced technologies and skills [4]. In fact, to engage social organizations to join in HECS has been a promising method to deliver HECS, such as life consulting services, mental care, healthcare, and sports activities.

However, a new problem raises regarding social organization participates in HECS, like the shortage of service staff and the low service quality, which mainly is due to the problematic cooperation among related stakeholders in HECS. Garzella and Fiorentino claimed that synergetic management may be a promising approach to optimize resource allocation, reduce information asymmetry, and thus improve service quality [5]. The idea of synergetic management originates from synergy theory which was first put forward by Hakon [6], who explained that in a complex and disorder system, subsystems are coupled to achieve order by internal and external interaction. From the perspective of synergy theory, conflicts and problems in social organization participating in HECS can be eased by optimizing the information subsystem, the resource subsystem, the supervision subsystem, the policy subsystem, and the stakeholder subsystem, thus to enhance social organization's motivation to engage in HECS and improve total social welfare [7-9].

There exists few studies discussing the synergetic development of social organizations participating in HECS in a qualitative way. In view of this, this paper explores the determinants of synergetic development in HECS with consideration of social organization by the path analysis. According to the analysis of HECS and social organization, a theoretical model is proposed from the external and internal perspective. External influencing factors contain supervision capacity and institutional climate; internal factors include stakeholder engagement, resource allocation, and information sharing. By path analysis, this paper finds that stakeholder engagement plays a critical role in synergetic development in HECS, resource allocation can only be improved by institutional climate, and supervision capacity cannot facilitate information sharing. This paper highlights the importance of stakeholders in HECS and first examines the impact of policy and supervision on HECS in a qualitative way.

The rest of the paper is organized as followed. In Section 2, this study deals with the literature review and proposes the conceptual framework and hypotheses. In Section 3, this study focuses on data and the research method. Data analysis and the results are shown in Section 4. In Section 5, this study discusses the results and implications. In Section 6, this study concludes the research and points out the research limitations.

Literature review and hypotheses

Literature review

Home-based elderly care service (HECS)

To address severe aging problems, HECS, which refers to professional cares provided at home to the elderly with formally assessed demands, has been widely applied [10, 11]. Recent studies in developing countries regarding HECS focused on conceptual definition [12], policy formulation [13],

operation mode, and service system design [14]. Developed countries have a relatively perfect HECS system with many studies on the input of service resources, independent elderly care, and smart elderly care [15, 16]. At the practical level, there are many good practical examples of HECS worldwide, like home assistance systems for elderly care in Sweden [17], the self-help and mutual aid for elderly care service in USA [18], and the ageing well program supported by Department for Work & Pensions in UK [19]. In China, Shanghai has been proposed that 90% of the elderly be taken care of by HECS [20]. Cities such as Beijing, Hangzhou, and Wuxi all have set goals for achieving the 90% elderly care service pattern by 2020 [21].

However, HECS still faces many challenges in China. On the one hand, if HECS is provided purely by the market, it is full of commercial motive and loses the characteristic of public welfare. On the other hand, it lacks sufficient funds and resources if being provided solely by the governments. The HECS has dual problems of market failure and government failure [22, 23]. There are some other problems like unimplemented associated systems and policies, insufficiency of social awareness and participation, ineffective supply and high vacancy of beds in nursing homes, and the shortage and low quality of industry personnel [21, 24].

Social organizations participating in HECS

In order to resolve the aforementioned problems, it is a must to encourage enthusiastic and capable social organizations to participate in HECS with multi-disciplinary cooperation of governments. Social organizations are non-profit institutions whose aim is not necessarily to provide shareholders a return on their investment but to achieve social outcomes through various projects and activities [25, 26]. Social organizations fulfill vital functions particularly for humanitarian-oriented activities that cannot be addressed by government departments and market institutions, such as health, non-formal education, relief, and capacity building, wildlife protection, and labor inclusion [8, 26, 27]. In HECS, social organizations typically participate in distinctive forms, such as specialized pension institutions, community units, volunteers, and charitable organizations. Specific operation modes include property company contracting service, inter-organizational resource sharing, and American Program of All-inclusive Care for the Elderly mode [28]. Social organizations participating in HECS aim at achieving public benefit, which allows them to increase the volume and quality of elderly care services and promote equality and satisfaction in the consumption of elderly care services [29, 30].

Because social organizations are non-profitable, they have to implement specific operation models and rely on multi-cooperation to accomplish their work [31]. The specific work of social organizations includes resource allocation, services supply, supervision, and so on, which is inevitable to contact governments, the elderly, insurance companies, financial institutions, and other

departments or individuals [32]. Hence, uncoordinated cooperation between social organizations and other subjects in the production of HECS may give rise to problems. Firstly, Jia et al. believe that the uneven spatial distribution and imbalance between supply and demand of resources make it difficult to meet the growing demand for diversified HECS [33]. Secondly, governments occupy dominant positions in HECS and lack the institutional guarantee and financial incentive for social organizations, resulting in the low motivation of social organizations to participate [34]. In addition, social organizations fail to establish a transparent system and regulation, and the personnel they employ normally have unsatisfactory professional quality, resulting in distrust in the public, especially to the elderly [21]. These problems are all triggered by the low collaborative level of the existing HECS. Therefore, exploring the synergetic development of social organizations participating in HECS is the key to improve the aging service industry in China.

Social organizations participating in HECS

The concept of synergy originated from enterprise management and its main purpose is to link all the activities of enterprises through cooperation so that the overall revenue is more than the sum of each sections' revenue [35]. Synergy is an effective way to improve management efficiency, optimize resource allocation, and improve service quality [5]. Existing studies about synergetic development mainly focus on public service, such as logistics transportation [36] and ecological protection [37].

Although it is one of the public services, there is not much research on the synergetic development of elderly care services. Existing research has focused on partnering relationships among social organizations to integrate different levels of health care services, thus providing low-cost care for the dying and promoting successful collaboration among them [38-40]. However, governments, communities, social organizations, the elderly, and other participants are more involved in the HECS with its higher requirements. There is still a lack of research on the specific paths of implementation to explore the synergetic development of social organizations participating in HECS.

According to Busetto et al., synergy is a feasible and sustainable method to cater to the growing demand for long-term care [9]. In this paper, the synergetic development of social organizations participating in HECS, which also means coordinated development or joined-up development [41], aims to provide a potential solution to problems raised by a fast-aging population. As a system, the synergetic development of social organizations participating in HECS contains several elements and processes. By coordinating internal demand with external climate, it is predicted to achieve desirable outcomes. The potential impacts of the system include better access to elderly care service, improved satisfaction and experience for the elderly, and high-efficient resource utilization [42]. In a word, it

is claimed to deliver elderly care service resources effectively and equitably, thereby improving social welfare.

Conceptual model and hypothetical relationships

It is challenging to find an abstract and conceptual perspective to analyze the synergetic development of social organizations participating in HECS. Social organization participating in HECS not only needs to schedule resource in different areas and share information among different parties, but also requires to coordinate benefits among different stakeholders including the government, the social organization, the community institution, and the elderly. Based on the case study, Ansell and Gash constructed a theoretical framework to analyze collaboration which starts from problem analysis [43]. By identifying the conflicts and constraints, they designed facilitative conditions and institutional measurements to optimize the collaborative process and achieve desirable outcomes. The basic constructs are helpful to analyze the synergetic development of social organizations participating in HECS [44]. In view of this, this paper explores external and internal influencing factors that may affect the synergetic development of social organizations participating in HECS. External influencing factors contains supervision capacity and institutional climate. Service supervision provides clear guidelines, such as service standards and improvement directions, to participants, and supervision capacity has an influence on the practical outcome of guidelines. Institutional climate acts as guidance and incentive to induce more elderly people as well as social organizations to engage in HECS. As for the internal factors, stakeholder engagement, resource allocation, and information sharing are examined. Stakeholders are the subjects of HECS. The degree of their engagement will influence the flow of resources and information in HECS. The resource is fundamental to provide HECS; while proper resource allocation is based on proper information sharing. Thus, stakeholders, resources, and information are all pivotal to facilitate the synergetic development of social organizations participating in HECS. The analysis framework is presented in Fig 1.

Fig 1. The analysis framework of synergetic development of social organizations participating in HECS

Resource allocation

HECS resource, including staff, funding, equipment, and so on, is significant for the elderly to attain efficient aging service [45]. If service supply is in short, aging service demand then cannot be well met. Similarly, the social aging service will achieve equity only if the resource is well allocated. Only if service resource is fully utilized can HECS be improved; Otherwise, some resources will be

wasted while some elderly people usually with low incomes cannot obtain care service. Therefore, we put forward:

H1. *Resource allocation is positively related to synergetic development.*

Information sharing

Information sharing means demand information as well as supply information are circulating in the aging service market. Information sharing plays an important role in service delivery and corporation[46, 47]. Accurate information and efficient transmission help the service-providers informed well about service demand, like service preference, current location, and so on[48]. Then service resources can be delivered to the right place. Besides, information sharing in one service chain can also trigger decision adjustments in another service chain, which has a direct impact on resource integration [46]. To learn about the demand of the elderly clearly and to share this information among service-providers timely and accurately are beneficial to service delivery as well as conducive to improving the elderly's sense of service contentment. Therefore, we put forward:

H2a. *Information sharing is positively related to resource allocation.*

H2b. *Information sharing is positively related to synergetic development.*

Stakeholder engagement

Stakeholder engagement refers to connection and corporation among different stakeholders in HECS. HECS involves four main stakeholders, namely, the government, the social organization, the community institution, and the elderly. In the traditional HECS model, the government is responsible for the total social aging service, from service production, service provision to service supervision, which has been proved to have low efficiency. Stakeholder engagement, however, could facilitate the resource and information to flow in the home-based care system [49]. By communication and interaction, resource allocation and information sharing are induced [50], thus promoting the sustainability of HECS. Therefore, we put forward:

H3a. *Stakeholder engagement is positively related to resource allocation.*

H3b. *Stakeholder engagement is positively related to information sharing.*

H3c. *Stakeholder engagement is positively related to synergetic development.*

Institutional climate

Institutional climate includes policy climate and market climate. The government has strong control over social resource due to its special status and has edges over making policy regarding service subsidies and nursery staffs to prosper the development of the aging service market [41]. Apart from the government, the market also plays a regulatory role in aging service delivery [51]. By setting clear service processes and the standard of quality, the market, to some extent, can overcome the problems of low service quality caused by information asymmetry. By clarifying the rights and

responsibilities of all parties in the market, communication and cooperation can be well promoted. The institutional climate is not only a constraint but also a support to facilitate service delivery. Therefore, we put forward:

H4a. *Institutional climate is positively related to resource allocation.*

H4b. *Institutional climate is positively related to information sharing.*

H4c. *Institutional climate is positively related to stakeholder engagement.*

H4d. *Institutional climate is positively related to synergetic development.*

Supervision capacity

Social organizations participating in HECS needs the constant exchange of resource and information among various elements to maintain and facilitate the development of HECS. One main aspect of supervision capacity is to ensure the availability of aging services [52]. In the process of resource allocation, supervision capacity induces resources to flow in different areas, thus promoting the optimal combination of resources and improving the fairness and effectiveness of resource allocation. The supervision mechanism also stimulates the circulation of home-based care information throughout the service network. Through social supervision and media supervision, service providers will strengthen communication and interaction among all parties. For example, by constructing a service feedback mechanism, service providers can gain a deeper understanding of service requirements or problems in the service chain, thereby improving service quality and satisfaction. Therefore, we put forward:

H5a. *Service supervision is positively related to resource allocation.*

H5b. *Service supervision is positively related to information sharing.*

H5c. *Service supervision is positively related to stakeholder engagement.*

H5d. *Service supervision is positively related to synergetic development.*

The above hypotheses are summarized in Fig 2.

Fig 2. Hypothesis for synergetic development of social organizations participating in HECS

Research method

Research design

In order to explore the extent to which determinants influence the synergetic development and the path that improves the synergetic development, the determinants, and hypothetical relationships were determined into the relation model based on the literature review and the characteristics of social organizations participating in HECS in China. In addition, the relevant items were identi[illegible]. A questionnaire survey using a stratified random sampling method was then conducted to collect the

data from the relevant elderly care organizations and agencies in Nanjing. Structural Equation Model (SEM) technique is a commonly used method to explain the hypotheses of relationships between observed variables and latent variables. To conduct the data analysis, it is by all means to establish two components: measurement model and the structural model by using SPSS 20.0 and AMOS 26.0 software. The measurement model is used to verify if latent variables are well measured by the observed variables, and the structural model is utilized to identify relationships among the latent variables. The research design is shown in Fig 3.

Fig 3. The research methodology

Measures

Since all variables are latent which cannot be directly measured, it is necessary to set the observation variables for measurement. Based on the previous literature, this study made some modifications according to its own actual conditions and developed its own measurement items. The measurement items and their sources are shown in Table 1.

Indicators measuring resource allocation

At present, most of the funding and resources mainly come from the Chinese government, which is far from meeting the demand. Exploring diversified funding sources of funds (RA1) and attracting sufficient resources (talent, facilities, funds) (RA2) will improve the satisfaction of all stakeholders [53]. In fact, synergetic development could also be improved through the properly designed allocation of resources standards (RA3, RA4) which cannot only trigger enthusiasm but also result in lowered total costs and higher efficiency.

Indicators measuring information sharing

Information sharing is fundamental to learn about other’s demands as well as attract other’s attention. In terms of social organizations participating in HECS, whether service providers know the demand of the elderly (IS2) [54], and the accuracy (IS3) [55] and efficiency (IS1) [54] of information sharing will influence the perception of service providers regarding service demand. Besides, information technology increases the availability of each participant for information sharing (IS5) [56].

Indicators measuring stakeholder engagement

For the relationship among stakeholders, the close engagement provides opportunities for stakeholders to communicate with each other in HECS. Therefore, the frequency of connection among four main stakeholders (SHC1, SHC2, SHC3, SHC4) reflects the degree, which will improve the efficiency of cooperation and service supply [57].

Indicators measuring institutional climate

Determining the status of the government is crucial in today’s environment because it can directly affect how the HECS industry is managed. Because of the dominant position of the government, it could contribute to a satisfactory institutional climate. Combined with the prior literature review, the factors influencing institutional climate can be described as follows. It was indicated that comprehensive policies, such as subsidy policies (IC1), professionals training policies (IC2), and clear standards (IC5), encourage social organizations into the HECS industry and facilitate its development [58, 59]. Clarifying the power and responsibility (IC3) among stakeholders is conducive to reducing the obstacles to the development of HECS, so as to further promote the legalization and institutionalization of HECS [60]. Rational entry and exit mechanisms (IC4) were considered as important factors to form an orderly competitive environment, which could benefit synergetic development [61].

Indicators measuring supervision capacity

Supervision in HECS requires the engagement of each stakeholder. For the social organization, a standard evaluation mechanism needs to be established (SC1) [62]. For the community institution, it has the closest relationship with the elderly and needs to check the service quality carefully (SC2). The government also needs to be responsible for the aging service market (SC3) [63]. As for the elderly, they care about whether their demand is well met (SC5) [64]. In addition, social media also plays an important role in service supervision (SC4) [63].

Indicators measuring synergetic development

The term synergy refers to combined, ‘cooperative’ effects that arise from the relationships and interactions among various stakeholders in a given context—effects that are not otherwise attainable. Synergetic development of social organizations participating in HECS means high-efficient resource utilization (SD1), better access to the service market (SD2), and improved satisfaction and experience for the elderly (SD3) [7, 9]. In a word, synergetic development is claimed to deliver aging services resource-effectively and equitably, thus improving social welfare (SD4). For instance, if resource utilization, market environment, and service satisfaction are compatible, the system would promote stakeholder cooperation and produce more social benefits.

Table 1. Measurement items of latent variables

Potential variables	Observation variables	Measurement items
Resource allocation	RA1	I think that stakeholders have different funding sources [53]
	RA2	I think there are sufficient resources (like facilities and funds) for stakeholders to use [53]
	RA3	I think that the allocation of resources (funds, facilities) helps to improve the efficiency of use [53]

	RA4	I think the allocation of resources (talents, facilities, funds) is fair [53]
Information sharing	IS1	I think the efficiency of information transmission (service demand, material allocation) among stakeholders is high [56]
	IS2	I think the symmetry of information (service demand, material allocation) among stakeholders is high [54]
	IS3	I think the accuracy of information (service demand, material allocation) transmission among stakeholders is high [55]
	IS4	I think the sharing degree of information (service demand, material allocation) among stakeholders is high [54]
	IS5	I think the application level of information technology is high[56]
Stakeholder engagement	SE1	I think I have a close engagement with (other) social organizations [57]
	SE2	I think I have a close engagement with (other) government departments [57]
	SE3	I think I have a close engagement with (other) community institutions [57]
	SE4	I think I have a close engagement with (other) the elderly [57]
Institutional climate	IC1	I think that there are comprehensive subsidy policies to encourage the participation of stakeholders [58]
	IC2	I think that there are comprehensive professionals training policies [59]
	IC3	I think that entry and exit mechanism for social organizations is rational [60]
	IC4	I think that power and responsibility mechanism for stakeholders is rational [61]
	IC5	I think that there are clear standards to regulate the service process and quality [61]
Supervision capability	SC1	I think there is a thorough performance evaluation system in social organizations [62]
	SC2	I think community institutions can effectively supervise social organizations and government departments [63]
	SC3	I think government departments can effectively fulfill their responsibilities (support, publicity, cooperation, and accountability) [63]
	SC4	I think the public and the media can actively participate and supervise [63]
	SC5	I think the opinions of the elderly can be effectively conveyed and implemented [64]
Synergetic Development	SD1	I think synergetic development will improve the effectiveness of stakeholders' expenditure [7, 9]
	SD 2	I think the popularization of synergetic development can attract more social organizations to participate [7, 9]
	SD 3	I think synergetic development will improve service satisfaction [7, 9]
	SD 4	I think synergetic development will improve social benefit [7, 9]

Data collection

The questionnaire is composed of two parts. The first part is the background information of the respondents, including the gender, age, education, working experience, and job category. The second part is the question of measuring the latent variables using the Likert seven-point scale ranging from 1 ("strongly disagree") to 7 ("strongly agree") measurement.

Before formal data collection, a pre-survey based on the questionnaire draft was conducted to ensure the consistency and accuracy of the questionnaires. With advice from 12 professionals, the

questionnaire was revised, improved, and validated. Then the formal questionnaire was conducted for people and agencies related to elderly services in Nanjing by means of random sampling. We received a total of 249 responses, and 211 were valid. The effective response rate was 84.74%, which was deemed adequate for the data analysis [65]. Among them, 80 questionnaires were from academic, 57 respondents were from the government department, 32 respondents were from service organizations, 42 respondents were from the elderly care association, which ensured the accuracy and scientificity of the results. More than 92% of the respondents have more than two years of elderly care work experience or research experience, which ensures the credibility and reliability of the questionnaire survey results. The background information of participants is presented in Table 2.

Table 2. Background information of participants

Demographics	Frequencies	Percentages (%)
**Gender**		
Male	114	54
Female	97	46
**Age**		
＜30	38	18
30-40	59	28
40-50	70	33
＞50	44	21
**Education level**		
High school	23	11
bachelor degree	38	18
Graduate degree	70	33
Ph.D. degree	80	38
**Job category**		
government department	57	27
Experts and scholars	80	38
Service organizations	32	15
Elderly care association	42	20
**Work experience (years)**		
＜2	15	7
2-5	59	28
5-8	84	40
＞8	53	25

Results

Measurement model

A confirmatory factor analysis was used to assess the reliability and validity of construct measures. Firstly, factor loadings were verified. Harman pointed out that the factor loadings should be above 0.7, which demonstrates that the indicator performs a satisfactory degree of reliability [66]. For this reason, two indicators (RA1 was 0.466, IT4 was 0.503) were deleted simultaneously and therefore CFA was a rerun. The final results are shown in Table 3. The CFA results suggested an

acceptable fit between the measurement model and data set, which shows Chi-square to the degree of freedom ($\chi2$/df) is 1.034, Comparative fit index (CFI) is 0.998, Goodness of fit index (GFI) is 0.901, Normed fit index (NFI) is 0.934, Incremental fit index (IFI) is 0.998 and Root mean square error of approximation (RMSEA) is 0.013.

Table 3. Results of confirmatory factors analysis

Constructs	Items	Factor loadings	Cronbach's alpha	CR	AVE
Institutional Climate	IC1	0.800	0.906	0.907	0.660
	IC2	0.822			
	IC3	0.822			
	IC4	0.819			
	IC5	0.798			
Stakeholder Engagement	SE1	0.846	0.898	0.898	0.687
	SE2	0.833			
	SE3	0.825			
	SE4	0.812			
Resource Allocation	RA2	0.826	0.864	0.871	0.691
	RA3	0.842			
	RA4	0.826			
Information Sharing	IS1	0.813	0.893	0.894	0.678
	IS2	0.821			
	IS3	0.835			
	IS5	0.824			
Supervision Capability	SC1	0.817	0.904	0.907	0.660
	SC2	0.825			
	SC3	0.824			
	SC4	0.820			
	SC5	0.775			
Synergetic Development	SD1	0.900	0.942	0.943	0.804
	SD2	0.892			
	SD3	0.905			
	SD4	0.889			

Note: CR=Composite Reliability, AVE=Average Variance Extracted

To assess the reliability of the constructs, Cronbach's Alpha values, and Composite Reliability (CR) were used. The values should be above 0.7 which shows the internal consistency of the construct is satisfactory [67]. As Table 3 shows, Cronbach's Alpha values ranged from 0.864 to 0.942, and CR ranged from 0.871 to 0.943, indicating good reliability for all of the constructs.

The convergent validity checks through the average variance extracted (AVE) [68]. As Table 3 shows, AVE ranged from 0.660 to 0.804, higher than the recommended threshold value of 0.50, indicating adequate convergent validity.

Structural model

A structural model was used to test the hypotheses. The output results of the fitting index of the model suggested the extended model had an acceptable model fit ($\chi2$/df=1.557, CFI=0.968,

NFI=0.915, IFI=0.968, RMSEA=0.052). Square multiple correlations (R2) can be used to measure the model's predictive accuracy [69]. Institutional climate, stakeholder engagement, resource allocation, information sharing, and supervision capability explained 87% of the variance of synergetic development.

Fig 4. The results of the proposed model

Table 4 shows the hypotheses testing results. First, we assessed the relationships between determinants and synergetic development and found institution climate ($\beta=.15$, $p<.05$), supervision capacity ($\beta=.24$, $p<.001$), stakeholder connection ($\beta=.28$, $p<.05$), information sharing ($\beta=.26$, $p<.05$) and resource allocation ($\beta=.21$, $p<.05$) had a significant impact on synergetic development.

Then we assessed the relationships among the five determinants. External influencing factors (institution climate and supervision capacity) were firstly tested. Institution climate had a significant impact on stakeholder connection ($\beta=.59$, $p<.001$), information sharing ($\beta=.24$, $p<.05$), and resource allocation ($\beta=.36$, $p<.001$). Supervision capacity also had a significant impact on stakeholder engagement ($\beta=.54$, $p<.001$) and resource allocation ($\beta=.29$, $p<.001$). However, there was no significant direct relationship between supervision capacity and information sharing ($\beta=.24$, $p>.05$), which failed to support H5b.

The relationship among internal influencing factors (stakeholder engagement, information sharing, and resource allocation) were tested lastly. Stakeholder engagement had a significant impact on information sharing ($\beta=.70$, $p<.001$) and had no significant direct impact on resource allocation ($\beta=.29$, $p>.05$), which supported H3b, not H3a. Information sharing had no significant direct impact on resource allocation($\beta=.134$, $p>.05$)，which failed to support H2a.

Table 4. Results of the structural equation modeling

Paths	Coefficients	t-values	Hypothesis	Result
RA→SD	0.207	2.358*	H1	Supported
IS→RA	0.134	0.917	H2a	Not Supported
IS→SD	0.256	2.346*	H2b	Supported
SE→RA	0.304	1.903	H3a	Not Supported
SE→IS	0.700	5.739***	H3b	Supported
SE→SD	0.284	2.340*	H3c	Supported
IC→RA	0.362	4.003***	H4a	Supported
IC→IS	0.244	2.757**	H4b	Supported
IC→SE	0.589	8.369***	H4c	Supported
IC→SD	0.147	2.051*	H4d	Supported
SC→RA	0.288	3.544***	H5a	Supported
SC→IS	0.01	0.120	H5b	Not Supported
SC→SE	0.544	8.024***	H5c	Supported
SC→SD	0.235	3.667***	H5d	Supported

Note: *$p<0.05$, **$p<0.01$, ***$p<0.001$, SD=Synergetic Development, RA=Resource Allocation, IS=Information Sharing, SE=Stakeholder Engagement, IC=Institutional Climate, SC=Supervision Capacity.

Discussion

This paper utilized an SEM model to show how variables may affect each other and contribute to synergetic efficiency. The relationships among six variables are presented in the model as shown in Figure 4 and Table 4. This paper discusses the relationships among internal variables (resource allocation, information sharing, and stakeholder engagement) and external variables (institutional climate, supervision capacity). Then the managerial implications are developed for the synergetic development of social organizations participating in HECS.

Improve synergetic development from an internal perspective

Resource allocation

The hypothesis was supported by the results of SEM, which means resource allocation has a positive influence on synergetic development (H1). The allocation of resources, including human resources, facilities, and equipment resource, and capitalresources, plays a pivotal role in the healthcare industry [70]. Resource allocation is ultimately a game among stakeholders. Hence, all stakeholders should jointly integrate related resources through a series of measures such as substitution, complementation, and introduction. In particular, government departments should guide social organizations to take these measures. Only in this way HECS can achieve the common goals of reducing the cost of resource distribution, rationalizing resource allocation, and improving the synergetic level.

Information sharing

According to the results of SEM, there are two hypothetical relationships about information sharing, one of which was significant and the other one was not. Information sharing is positively related to synergetic development (H2b). According to Lotfi et al., information sharing enables stakeholders to obtain all kinds of required information [71]. The various elements, such as resource allocation, institution design, and service supervision, are organically linked through information flow to cooperate and support each other, so as to eliminate the isolation of stakeholders and realize the multiple combinations of obligations, responsibilities, and benefits.

However, information sharing is not positively related to resource allocation (H2a). The insignificance of this hypothesis can be explained from both macro and micro perspectives. From a macro perspective, the shared information, such as favorable policy orientation or market demand, will prompt more social organizations to enter the elderly care services industry [72]. However, as the resources of the whole industry are relatively limited at present [10], social organizations will plunder these resources, leading to the phenomenon of unfair resource allocation. From a micro perspective, social organizations in Chinese aging services industry are also in a state of resource

shortage at present [73], so the sharing of such information is of no help. For example, although all the social organizations know that there is a lack of professional talent, such shared information has not significantly improved the allocation of talent resources, and the industry still faces the problem of a talent shortage for a long time. There has always been a serious shortage of related resources in aging services industry [74], and only by solving the problem of resource allocation can we fundamentally solve the problem of information sharing.

Stakeholder engagement

As presented before, there are three hypothetical relationships among stakeholder engagement and other variables. Firstly, stakeholder engagement does not have a significant influence on resource allocation (H3a). This result may be related to the insignificance of H2a analyzed above. Stakeholder engagement can promote information sharing, but information sharing does not promote resource allocation, so stakeholder engagement may not improve resource allocation. As one of the most intractable problems in aging service industry, the question that how to allocate resource should be determined by institutions and supervision, rather than stakeholders who are self-interested.

Secondly, stakeholder engagement is positively related to information sharing (H3b) with standardized coefficient values of 0.700, which is the highest value among all the hypotheses. Connection and corporation among stakeholders can integrate professional work in elderly care services, thus breaking down information communication barriers in various work and improving the operation efficiency of HECS.

Thirdly, H3c has a standardized coefficient value of 0.284, which means stakeholders should take responsibility and maintain cooperation to promote synergetic development. The relationship among stakeholders of HECS is interdependent and mutually beneficial, which means the survival and development of a single subject in the system cannot be separated from the support and promotion of other subjects [75]. According to their own interests and the overall interests of HECS, the stakeholders shall formulate feasible and mutually beneficial strategies, and dynamically adjust them according to the external environment and the development of HECS, so as to jointly participate in the market competition and give full play to their respective comparative advantages.

Improvement of synergetic development from an external perspective

Institutional climate

As presented before, there are four hypothetical relationships among institutional climate and other variables. The four hypotheses are supported by the results of SEM, which means the institutional climate is the fundament to improve the synergetic development of social organizations

participating in HECS. First, the institutional climate is positively associated with resource allocation (H4a) with standardized coefficient values of 0.362. This result verifies that institutional forces of legitimacy are not iron cages and a fair endowment policy and reasonable competitive market environment tend to integrate idle social resources, improve endowment resource utilization efficiency and improve the satisfaction of the elderly [76]. The results are consistent with the findings of Garg etc., which revealed policies can ensure optimal allocation of scarce healthcare resources [77]. Second, the relationship between institutional climate and information sharing (H4b) was verified. The institutional climate is significantly associated with information sharing with standardized coefficient values of 0.244. It is easy to understand that institutional guarantee will provide information exchange platforms and construct perfect information communication channels for stakeholders in the elderly care industry to provide support across the continuum of care [78]. Third, institutional climate positively relates to stakeholder engagement (H4c) with the highest standardized coefficient value of 0.589. It is indicated that the institutional climate is significantly associated with stakeholder engagement, which is an important antecedent. In other words, a fair endowment policy and a reasonable competitive market are the key factors to induce stakeholder positive engagement and clarify the rights and responsibilities [79]. In this case, stakeholders give full play to their advantages to form a good industrial order. H4d was set to show that institutional climate directly influences synergetic development, whose path is weak with a standardized coefficient value of 0.147. This is because the relationship between institutional climate and synergetic development is partially mediated by affecting stakeholder connection. Therefore, the path of institutional climate and synergetic development is still significant.

Supervision capacity

There are four hypothetical relationships among supervision capacity and other internal variables, three of which were supported by the results of SEM. First, supervision capacity remains significantly associated with resource allocation (H5a) with standardized coefficient values of 0.288. The efficient supervision strategy of government authorities and pension institutes not only minimizes waste of resources but also helps in identifying, as well as exploiting idle resources available in the HECS system [80]. Resources integrated during such supervision could greatly improve elderly care service supplier’s overall efficiency. Second, supervision capacity was not found to have a significant positive influence on information sharing. Two different explanations for the non-significance of this hypothesized effect were posed. On the one hand, it seems that supervision standards of the elderly-care industry are not comprehensive to deal with information accessed from diversified channels. For example, supervision standards on social networks are missing, which prevents access to high-quality feedback information from the elderly. On the other hand, it seems that even though the information

has been widely supervised by home-based elderly care platforms, it does not mean that the means of information sharing are efficient [81]. Third, the result for hypothesis H5c shows that service supervision is highly beneficial for stakeholder engagement with the highest standardized coefficient values of 0.544. Multi-stakeholder supervision systems in the pension industry could focus on jointly working and providing knowledge between government and social organizations, regularly communicating with the elderly, but also are still valuable in developing long-term cooperation. On the basis of the theoretical findings, H5d has the lower standardized coefficient value of 0.235, which means supervision capacity has little influence on the synergetic development. de Graaff etc. emphasized the need for pragmatic and reflexive engagement with clients in the supervision of long-term elderly care [82]. Therefore, communication barriers are a major obstacle to effective supervision. Roberts etc. promoted social media which could be used to an effective way to supervision, which not only stimulates the participation of the elderly but also improves the quality and safety of care service [83].

Implications

Collaboration, communication, and coordination are of great importance to the synergetic development of social organizations participating in HECS. To enhance collaboration, stakeholder engagement is fundamental [49]. Meanwhile, governments play a guidance role in the development of HECS [84], who are capable to launch several incentive policies, such as subsidy mechanisms and staff introduction strategies, to induce service providers including social organizations and community institutions to participate in the aging industry. It is also a must for governments to clarify the duty of each party and the standard of service delivery via policy and rules. As for service providers, they could form an alliance to deal with some difficulties, such as resource shortage and communication barriers. They could also build a platform to strengthen the bonds among different service suppliers and service demanders. As a whole, it is important for service providers to innovate the model of service provision with the help of technology [85]. As far as the elderly are concerned, their awareness of using aging care services should be aroused by the public, and they should change their traditional views towards aging and adapt to HECS. Institutes for senior citizens can be constructed to improve the elderly’s living skills and consumer awareness. Moreover, young citizens should also be included in the elderly care service by some activities like time bank, to cultivate their consumption awareness regarding aging service in advance.

To improve communication, information sharing, institutional guarantee, and service supervision are equally important. With the growing aging population, a demand database is helpful to store and deal with numerous demand information. By artificial intelligence and big data, changes in demand can be captured timely and accurately, then personalized service could be delivered to each

old man. The institutional guarantee means that the government could discover some aging problems via policies and remind service providers of some business opportunities. The service feedback mechanisms can also help service providers improve the service quality as well as keep pace with the changes in the service demand. Apart from learning about service demand, a compatible and public service system is helpful to reduce repetitive work and improve service efficiency [86].

Finally, to achieve coordination, public-private-partnership are suggested in HECS [87]. By cooperation between governments and social organizations, resource shortage, information barriers, and some other problems can be alleviated. By delegating social organization to establish digital systems and technological infrastructures, governments not only provide the social organization with business chances but also manage the aging industry in a good manner.

Conclusion

This study investigated the determinants of the synergetic development of social organizations participating in HECS. These determinants can be divided into two external variables and three internal variables. Through the literature review, this study explored the indicators of these determinants. The data on the determinants and indicators were collected from elderly agencies and research agencies in Nanjing, China. A conceptual model was proposed to explain the relationships between synergetic development and these determinants. An SEM was conducted to test the proposed conceptual model and identify the major determinants affecting the synergetic development of social organizations participating in HECS.

The results of the SEM indicate all the determinants have a positive direct impact on the synergetic development of social organizations participating in HECS. The nine hypothetical relationships among external variables and internal variables were tested by the SEM method. Most hypothetical relationships were positively supported except H2a, H3a, H5b. The results indicated the direction of stakeholder engagement on the synergetic development were identified to be more important than other determinants. In addition, five determinants can indirectly influence the synergetic development by affecting other determinants. Furthermore, several useful suggestions can be drawn from the research findings from the perspective of government, social organizations, community, and elderly people.

The identified determinants and relationships would be rather useful for the synergetic development of social organizations participating in HECS. It can effectively enhance the participation of social organizations and improve the efficiency of the elderly care industry. Although this study obtained useful findings regarding institutional climate, supervision capacity, stakeholder engagement, information sharing, and resource allocation, more determinants should be investigated by literature review and the actual situation. Moreover, their relationships should be clarified by

further research. More indirect influence on synergetic development should be investigated in the future. In addition, more data and information should be stratified analysis based on government, social organizations, community, and elderly people to avoid some deviations.

Conflict of Interest: The authors declare that they have no conflict of interest.

Acknowledgments

The authors’ special thanks go to all reviewers of the paper and to the National Natural Science Foundation of China (71671042), National Social Science Foundation of China (19CGL065) and the Fundamental Research Funds for the Central Universities for financially supporting this research.

References

1. Statistics NBo. China Statistical Yearbook 2019: The National Bureau of Statistics of China; 2019 [cited 1 May 2020]. Available from: http://data.stats.gov.cn/easyquery.htm?cn=C01.
2. Yan BQ, Gaol XL, Shen ZJ. Prospective living arrangement of China's urban elderly and development of an Agent-based Simulation (ABS) model for elderly care needs. Int Rev Spat Plan Sustain Dev. 2018;6(1):63-82.
3. Yuan JF, Li LZ, Wang ED, Skibniewski MJ. Examining sustainability indicators of space management in elderly Facilities-a case study in China. J Clean Prod. 2019;208:144-59.
4. Onunkwor OF, Al-Dubai SAR, George PP, Arokiasamy J, Yadav H, Barua A, et al. A cross-sectional study on quality of life among the elderly in non-governmental organizations' elderly homes in Kuala Lumpur. Health Qual Life Outcomes. 2016;14:10.
5. Garzella S, aele Fiorentino R. Synergy Value and Strategic Management: Springer; 2017.
6. Haken H. Synergetics. Europhysics News. 1976;7(7-8):9-12.
7. Tieman J, Mitchell G, Shelby-James T, Currow D, Fazekas BS, O'Doherty LJ, et al. Integration, coordination and multidisciplinary approaches in primary care: a systematic investigation of the literature. 2017.
8. Gunawan AS, Fiarni C, Ryana Y, editors. Design for performance monitoring system using earned value analysis method for nonprofit organizations case study of organization X, Indonesia. 2017 3rd International Conference on Science in Information Technology (ICSITech); 2017: IEEE.
9. Busetto L, Luijkx K, Vrijhoef HJM. Development of the COMIC Model for the comprehensive evaluation of integrated care interventions. Int J Care Coord. 2016;19(1-2):47-58.
10. Suurmond J, Rosenmöller DL, el Mesbahi H, Lamkaddem M, Essink-Bot M-L. Barriers in access to home care services among ethnic minority and Dutch elderly–A qualitative study. Int J Nurs Stud. 2016;54:23-35.
11. Henderson EJ, Caplan GA. Home sweet home? Community care for older people in Australia. J Am Med Dir Assoc. 2008;9(2):88-94.
12. Cao M, editor The Concept of Defining Home Care for the Aged Services Based on the Connotation and Extension in China. Proceedings of 2015 2nd International Conference on Industrial Economics System and Industrial Security Engineering; 2016: Springer.
13. Ye L-e, Qi Y-n, Ren H, editors. Research on China's Policies on Intelligent Services for the Aged Based on the Perspective of Policy Tools. 4th International Conference on Contemporary Education, Social Sciences and Humanities (ICCESSH 2019); 2019: Atlantis Press.
14. Chen Y-Y, Chen H, Song P. Promises and pitfalls of integrating home-based health services into Shanghai's elder-care system. Ageing & Society. 2018:1-21.
15. Davies B, Challis D. Matching resources to needs in community care: An evaluated demonstration of a long-term care model: Routledge; 2018.
16. Dzissah DA, Lee J-S, Suzuki H, Nakamura M, Obi T. Privacy Enhanced Healthcare Information Sharing System for Home-Based Care Environments. Healthc Inform Res. 2019;25(2):106-14.
17. Bergman MA, Johansson P, Lundberg S, Spagnolo G. Privatization and quality: Evidence from elderly care in Sweden.

J Health Econ. 2016;49:109-19.

18. Kyrouz EM, Humphreys K, Loomis C. A review of research on the effectiveness of self-help mutual aid groups. British J Clin Psychol. 2002;33:198-200.
19. Bowling A. Ageing well: Quality of life in old age: McGraw-hill education (UK); 2005.
20. Li H, Wang J, Zhang R, editors. Study on the financial investment level of home care services in Shanghai. 2017 29th Chinese Control And Decision Conference (CCDC); 2017: IEEE.
21. Bian S, Guo Q, Wang X. Construction of senior service system in the context of a healthy China. Global Health Journal. 2018;2(4):28-34.
22. Shuyu L. The Function of the Government, Market, and Family in the Elderly Long-term Care Insurance in China. Int J Bus Soc Sci. 2016;7(9).
23. Yazbeck AS, Soucat A. When both markets and governments fail health. Health Systems & Reform. 2019;5(4):268-79.
24. Hunter NJ, Wells Y, Clune SJ, Quintanilla BPA, Johnstone E. Choice and quality in home-based and community-based aged care: insights from two rapid evidence reviews. Ageing & Society. 2019:1-42.
25. Demerjian P, Lewis-Western M, McVay S. How does intentional earnings smoothing vary with managerial ability? Journal of Accounting, Auditing & Finance. 2020;35(2):406-37.
26. Moczulska M, Seiler B, Stankiewicz J. Coopetition in for-profit and non-profit organizations-micro level. Manage. 2019;23(2):138-56.
27. Nanthagopan Y, Williams N, Thompson K. Levels and interconnections of project success in development projects by Non-Governmental Organisations (NGOs). Int J Manag Proj Bus. 2019.
28. Hansen JC, Hewitt M. PACE provides a sense of belonging for elders. Generations. 2012;36(1):37-43.
29. Pestoff V. Co-production and third sector social services in Europe: Some concepts and evidence. Voluntas: International Journal of Voluntary and Nonprofit Organizations. 2012;23(4):1102-18.
30. Osborne SP, Radnor Z, Strokosch K. Co-production and the co-creation of value in public services: a suitable case for treatment? Public Manag Rev. 2016;18(5):639-53.
31. Ceptureanu SI, Ceptureanu EG, Bogdan VL, Radulescu V. Sustainability perceptions in Romanian non-profit organizations: an exploratory study using success factor analysis. Sustainability. 2018;10(2):294.
32. Yu J, Qu Z. PPPs: Inter-actor relationships two cases of home-based care services in China. Public Administration Quarterly. 2012:238-64.
33. Jia M, Zhou Y, Lin J. The Privatisation and the Unbalanced Spatial Development of Residential Care for the Elderly: the Case of Beijing, China. Appl Spat Anal Policy. 2018;11(1):59-80.
34. Montin S. Local government and the market. The case of public services and care for the elderly in Sweden. Public and Social Services in Europe: Springer; 2016. p. 87-101.
35. Haken H. Synergetics: Introduction and advanced topics: Springer Science & Business Media; 2013.
36. Sun Q, Wang X, Ma F, Han Y, Cheng Q. Synergetic Effect and Spatial-Temporal Evolution of Railway Transportation in Sustainable Development of Trade: An Empirical Study Based on the Belt and Road. Sustainability. 2019;11(6):1721.
37. Qiao J, Wang M, Zhang D, Ding C, Wang J, Xu D. Synergetic development assessment of urban river system landscapes. Sustainability. 2017;9(12):2145.
38. Booth M, Hill G, Moore MJ, Dalla D, Moore M, Messenger A. The new Australian Primary Health Networks: how will they integrate public health and primary care. Public Health Res Pract. 2016;26(1).
39. Shah BK, Shah T. Home hospice care in Nepal: a low-cost service in a low-income country through collaboration between non-profit organisations. The Lancet Global Health. 2017;5:S19.
40. Tong CE, Franke T, Larcombe K, Sims Gould J. Fostering inter-agency collaboration for the delivery of community-

based services for older adults. British Journal of Social Work. 2018;48(2):390-411.
41. Exworthy M, Powell M, Glasby J. The governance of integrated health and social care in England since 2010: great expectations not met once again? Health Policy. 2017;121(11):1124-30.
42. Mason A, Goddard M, Weatherly H, Chalkley M. Integrating funds for health and social care: an evidence review. J Health Serv Res Policy. 2015;20(3):177-88.
43. Ansell C, Gash A. Collaborative Governance in Theory and Practice. J Public Adm Res Theory. 2007;18(4):543-71.
44. Emerson K, Nabatchi T, Balogh S. An Integrative Framework for Collaborative Governance. J Public Adm Res Theory. 2011;22(1):1-29.
45. Owili PO, Hsu YH, Chern JY, Chiu CH, Wang B, Huang KC, et al. Perceptions and attitudes of health professionals in kenya on national health care resource allocation mechanisms: a structural equation modeling. PLoS One. 2015;10(6):e0127160.
46. Guan Z, Zhang X, Zhou M, Dan Y. Demand information sharing in competing supply chains with manufacturer-provided service. Int J Prod Econ. 2020;220:107450.
47. Osei-Frimpong K, Wilson A, Lemke F. Patient co-creation activities in healthcare service delivery at the micro level: The influence of online access to healthcare information. Technol Forecast Soc Change. 2018;126:14-27.
48. Woodward CA, Abelson J, Tedford S, Hutchison B. What is important to continuity in home care? Social Science & Medicine. 2004;58(1):177-92.
49. Dahan S, Bourque CJ, Reichherzer M, Ahmed M, Josee P, Mantha G, et al. Beyond a Seat at the Table: The Added Value of Family Stakeholders to Improve Care, Research, and Education in Neonatology. J Pediatr. 2019;207:123-9 e2.
50. Ehrenhard M, Kijl B, Nieuwenhuis L. Market adoption barriers of multi-stakeholder technology: Smart homes for the aging population. Technol Forecast Soc Change. 2014;89:306-15.
51. Go Jefferies J, Bishop S, Hibbert S. Customer boundary work to navigate institutional arrangements around service interactions: Exploring the case of telehealth. J Bus Res. 2019;105:420-33.
52. Levenson SA. The impact of laws and regulations in improving physician performance and care processes in long-term care. J Am Med Dir Assoc. 2004;5(4):268-77.
53. Owili PO, Hsu Y-HE, Chern J-Y, Chiu C-HM, Wang B, Huang K-C, et al. Perceptions and attitudes of health professionals in kenya on national health care resource allocation mechanisms: a structural equation modeling. PloS one. 2015;10(6).
54. Kajonius PJ, Kazemi A. Structure and process quality as predictors of satisfaction with elderly care. Health Soc C Community. 2016;24(6):699-707.
55. Tuinman A, de Greef MH, Krijnen WP, Paans W, Roodbol PF. Accuracy of documentation in the nursing care plan in long-term institutional care. Geriatric nursing. 2017;38(6):578-83.
56. Kuo M-H, Wang S-L, Chen W-T. Using information and mobile technology improved elderly home care services. Health Policy Technol. 2016;5(2):131-42.
57. Yang D, He Q, Cui Q, Hsu S-C. Non-economic motivations for organizational citizenship behavior in construction megaprojects. Int J Proj Manag. 2020;38(1):64-74.
58. Hladka M, Hyanek V, Spalek J. The Relationship between Public Subsidies and Unearned Revenues for Non-profit Organizations: Testing the Crowding-Out and Crowding-In Positions in the Czech Republic. Ekonomicky Casopis. 2017;65(3):263-81.
59. Sunde OS, Oyen KR, Ytrehus S. Do nurses and other health professionals' in elderly care have education in family nursing? Scand J Caring Sci. 2018;32(1):280-9.
60. Tokunaga M, Hashimoto H. Factors affecting the entry of for-profit providers into a price regulated market for formal long-term care services: A case study from Japan. Soc Sci Med. 2013;76:143-9.

61. Rasmussen B. Home-based care as a greedy organisation. Power and responsibility in decentralised organisations. Tidsskr Samfunnsforsk. 2000;41(1):38-57.
62. Bing-Jonsson PC, Hofoss D, Kirkevold M, Bjørk IT, Foss C. Sufficient competence in community elderly care? Results from a competence measurement of nursing staff. BMC nursing. 2016;15(1):5.
63. Shen J, Tang S, Xu C. Analysis and research on home-based care for the aged based on insurance policy under government leading. AMSE Journals-AMSE IIETA-Series: Advances A. 2017;54(1):106-26.
64. Kazemi A, Kajonius P. Variations in user-oriented elderly care: a multilevel approach. Int J Qual Serv Sci. 2017.
65. Babble E, MOUTON J. The practice ofsocial research. Southern Africa, Cape Town: Oxford University Press; 2003.
66. Harman HH. Modern factor analysis: University of Chicago press; 1976.
67. Lopes JRN, de Araújo Kalid R, Rodríguez JLM, Ávila Filho S. A new model for assessing industrial worker behavior regarding energy saving considering the theory of planned behavior, norm activation model and human reliability. Resour Conserv Recycl. 2019;145:268-78.
68. Chiu C-M, Wang ET. Understanding Web-based learning continuance intention: The role of subjective task value. Inf Manage. 2008;45(3):194-201.
69. Hu Lt, Bentler PM. Cutoff criteria for fit indexes in covariance structure analysis: Conventional criteria versus new alternatives. Structural equation modeling: a multidisciplinary journal. 1999;6(1):1-55.
70. Speziale G. Strategic management of a healthcare organization: engagement, behavioural indicators, and clinical performance. European Heart Journal Supplements. 2015;17(suppl_A):A3-A7.
71. Lotfi Z, Mukhtar M, Sahran S, Zadeh AT. Information Sharing in Supply Chain Management. Procedia Technology. 2013;11:298-304.
72. Kim S, Lee H. The impact of organizational context and information technology on employee knowledge‐sharing capabilities. Public Adm Rev. 2006;66(3):370-85.
73. Shen R-q, Zhou J. Research on the Problem of Urban Community-based Elderly Care Service under the Background of Aging Population-The Case Study of G City. DEStech Transactions on Environment, Energy and Earth Sciences. 2016;(peee).
74. Wang L, He L. Living arrangements and support resources for China’s elderly: findings from CLHLS 2018. China Population and Development Studies. 2020;3(4):310-26.
75. Rusconi G. Ethical firm system and stakeholder management theories: a possible convergence. Eur Manag Rev. 2019;16(1):147-66.
76. Kokkonen K, Rissanen S, Hujala A. The match between institutional elderly care management research and management challenges - a systematic literature review. Health Res Policy Syst. 2012;10.
77. Garg L, McClean S, Meenan B, Millard P. A non-homogeneous discrete time Markov model for admission scheduling and resource planning in a cost or capacity constrained healthcare system. Health C Manage Sci. 2010;13(2):155-69.
78. Song PP, Chen Y. Public policy response, aging in place, and big data platforms: Creating an effective collaborative system to cope with aging of the population. Bioscience Trends. 2015;9(1):1-6.
79. Setia M, Islam AM, Thompson JP, Matchar DB. Stakeholders' perspective on issues and challenges associated with care and treatment of aging-related cognitive impairment disorders in Singapore. Int Psychogeriatr. 2011;23(9):1421-32.
80. Yamashita T, Jeon H, Bailer AJ, Nelson IM, Mehdizadeh S. Fall Risk Factors in Community-Dwelling Elderly Who Receive Medicaid-Supported Home- and Community-Based Care Services. J Aging Health. 2011;23(4):682-703.
81. Batet M, Isern D, Marin L, Martinez S, Moreno A, Sanchez D, et al. Knowledge-driven delivery of home care services. J Intell Inf Syst. 2012;38(1):95-130.
82. de Graaff MB, Stoopendaal A, Leistikow I. Transforming clients into experts-by-experience: A pilot in client

participation in Dutch long-term elderly care homes inspectorate supervision. Health Policy. 2019;123(3):275-80.
83. Roberts MA, Williams BO. Medical supervision of frail elderly people in long term care. Scott Med J. 1997;42(5):133-4.
84. Liu L, Stroulia E, Nikolaidis I, Miguel-Cruz A, Rios Rincon A. Smart homes and home health monitoring technologies for older adults: A systematic review. Int J Med Inform. 2016;91:44-59.
85. Stoop A, de Bruin SR, Wistow G, Billings J, Ruppe G, Leichsenring K, et al. Exploring improvement plans of fourteen European integrated care sites for older people with complex needs. Health Policy. 2019;123(12):1135-54.
86. Xiong W, Chen B, Wang H, Zhu D. Governing public-private partnerships: a systematic review of case study literature. Aust J Public Adm. 2019;78(1):95-112.

Click here to access/download;Figure;Fig 1..tif

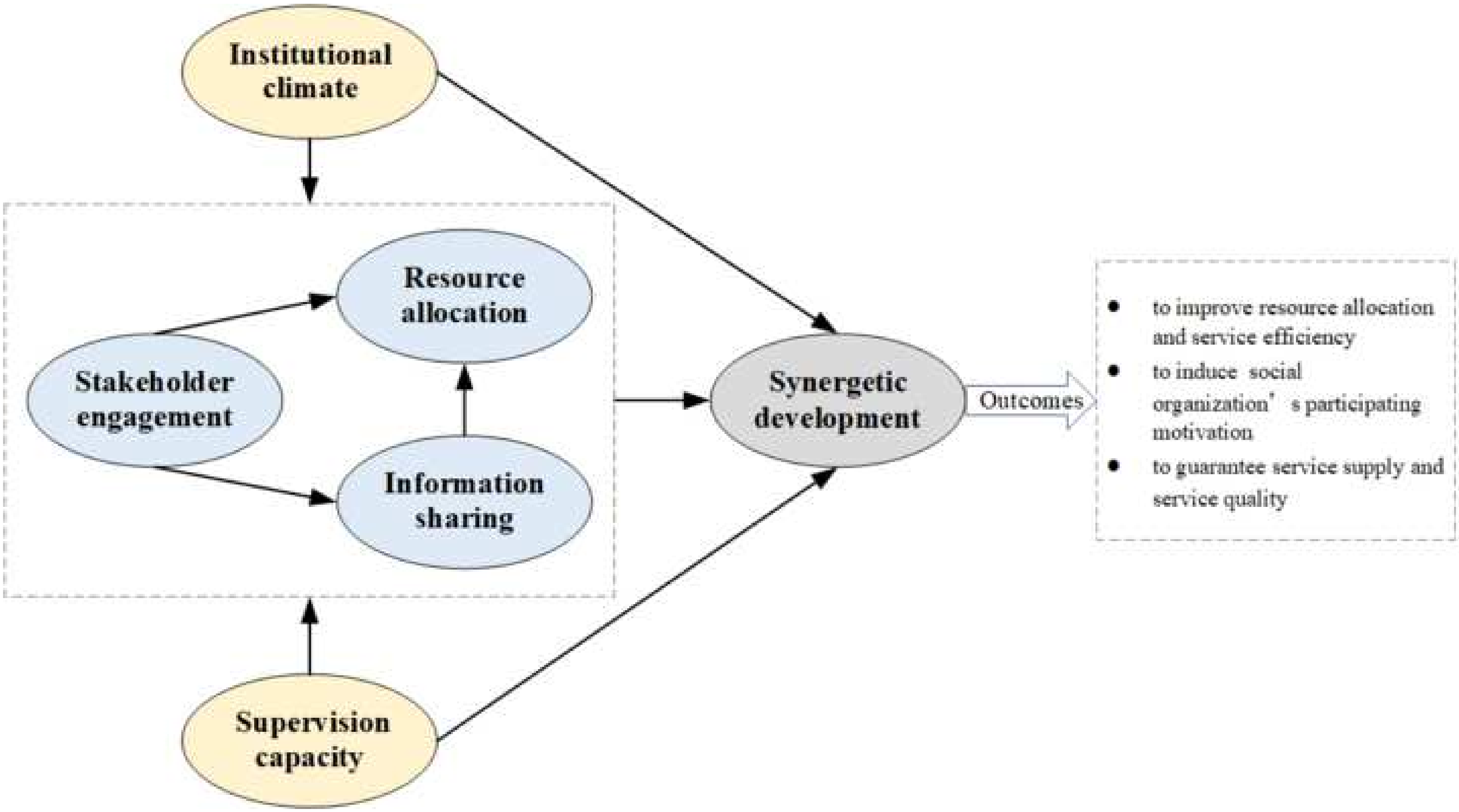

Fig 2

Click here to access/download;Figure;Fig 2..tif

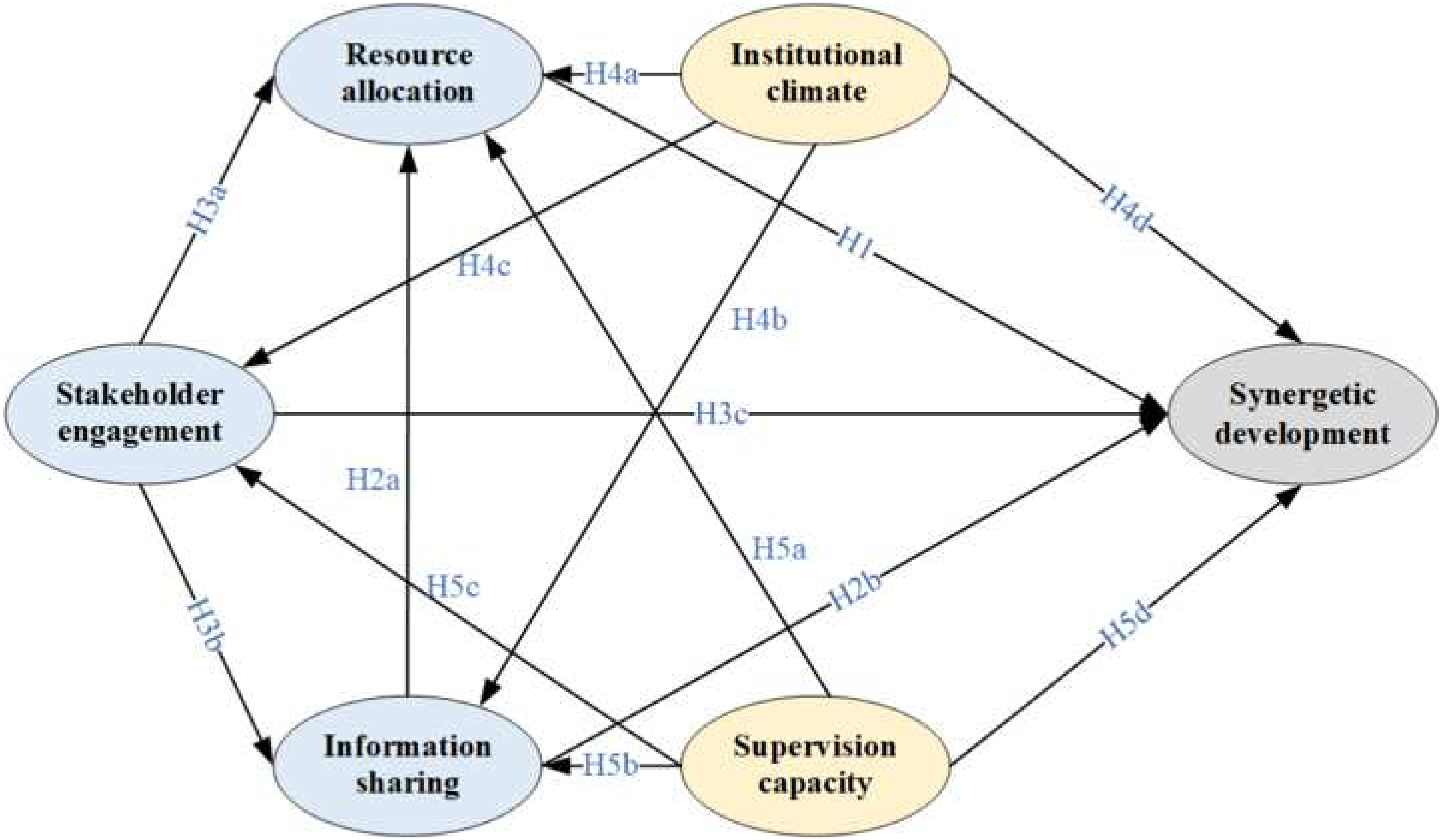

Click here to access/download;Figure;Fig 3..tif

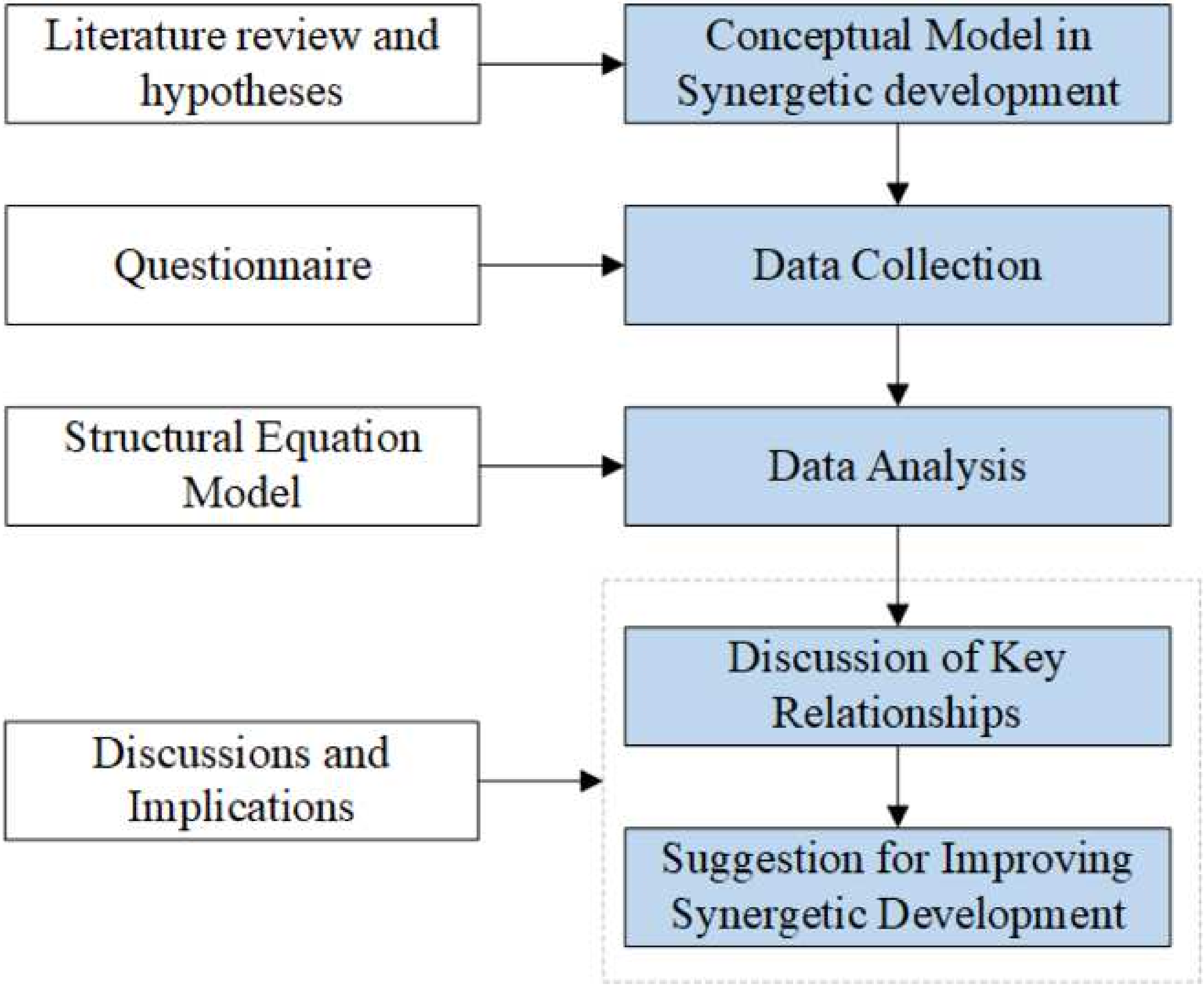

Fig 4

Click here to access/download;Figure;Fig 4..tif

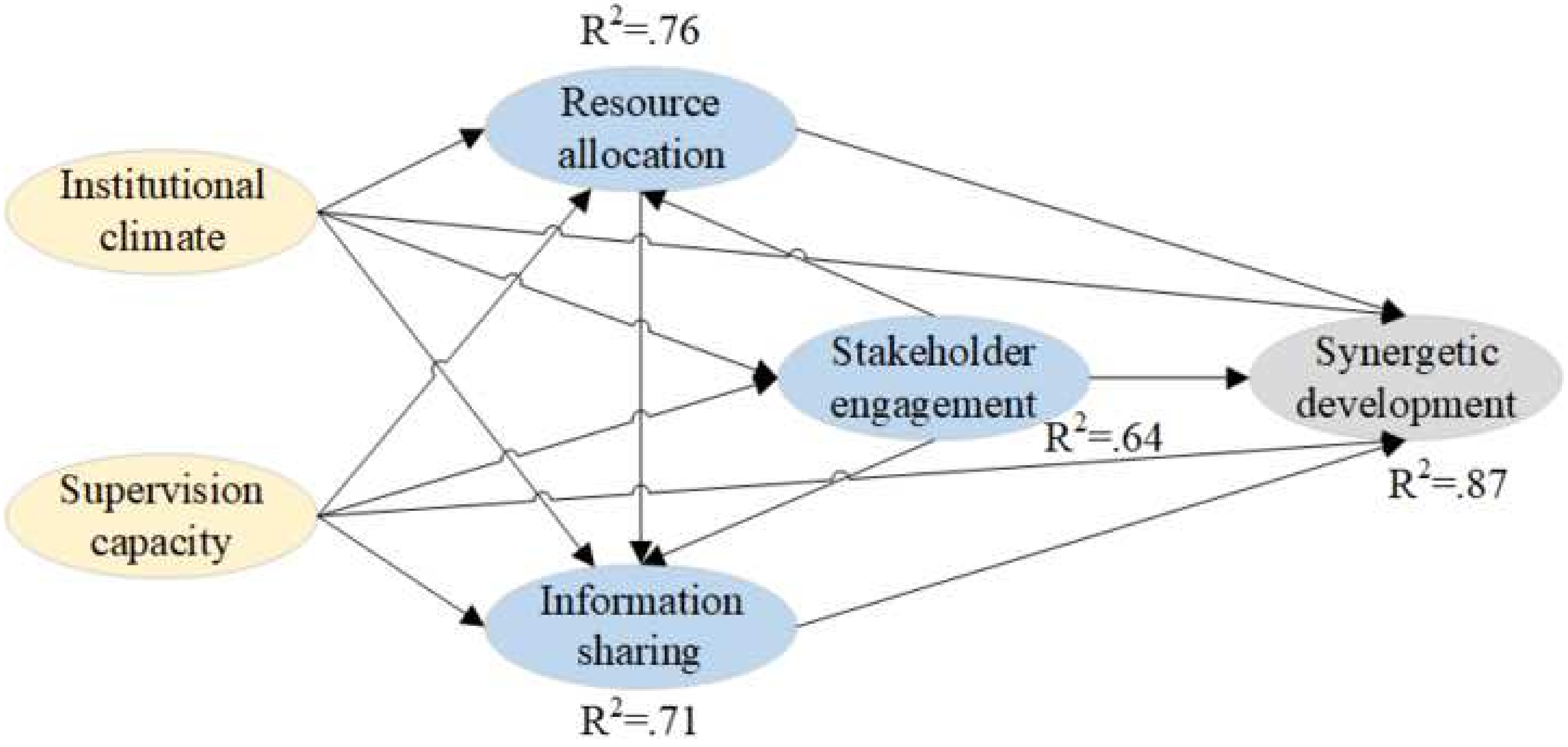

Click here to access/download
Supporting Information
DATA & INDICATORS.xlsx
