# Supplementary material for: Exploring the determinants of synergetic development of social organizations participating in home-based elderly care service: An SEM method
Source: PLoS One. 2020 Dec 31;15(12):e0244880. doi: 10.1371/journal.pone.0244880 (PMC7775099; doi:10.1371/journal.pone.0244880)
Supplement: Attachment — Submitted filename: reviewer comments.docx [file pone.0244880.s003.docx]

**Review Report** (PONE-D-20-20452)

Ref: (PONE-D-20-20452)
Title: Exploring the Determinants of Synergetic Development of Social Organizations Participating in Home-based Elderly Care Service: An SEM Method
Journal: PLOS ONE

I've read with attention this paper and I believed that this study could be of interest to many readers and helpful for health policy makers. synergy is one of the main components in high level of systems theory, and it can increase synergistic thoughts and actions in workplace environment. This study explores determinant factors on synergetic management in home-based elderly care service using a literature review, and then examines the relationship between variables using structural equation model. However, I believed that this study has many flaws and need a major revision.

1. Abstract:

The abstract of the paper is not clearly written. In abstract, background, objective, methods, results and conclusion must be summarized. But, in this abstract we can not find the purpose of the study and conclusion. Also, the methodology of this research in abstract is not clearly. I recommend that the keywords revised based on MeSH database.

2. Introduction:

Introduction starts well and we get acquainted with changes in population structure and changes in family structure. These changes create a demand for home-based elderly care services. But it does not continue as well. Introduction does not have a proper hierarchy. It is better that you review the previous work similar to your research in introduction. Also, many of content in literature review section is repeated. I suggest that literature review can be merge into introduction. I fined two heading title “Social organizations participating in HECS” in literature review.

However, the biggest problem with the introduction is that you have included factors influencing synergetic management in the introduction.

The other problem is that conceptual model and hypothetical relationships should be transferred to the method and results section.

First, you have to explain how choose the determinant factors. What search strategy were used and which databases did you search?

3. Methods:

SEM is a powerful technique to test and evaluate multivariate relationships.

The main problem in this study is related to methods. methodology of the paper needs more elaboration and clarity. I recommended that rewrite the methods of your manuscript and explain the methodology step-by-step. How you selected the variables affecting on synergetic management?

How you selected the measurement items and design the questionnaire?

Also, please provide the steps of SEM model in your research clearly.

Table 2 and sociodemographic characteristics of participants should be presented in the results section.

4. Results:

Many contents of results should have been presented in the method section (such as line 325-327, 336-337 and 340). Overall results are good.

5. Discussion:

The discussion is the repeat of results and justifications have not been given on how and why current study results are different or alike to the past studies. I recommend that to support the discussion based on the reported results with the past researches, so that it clearly justify present research.

6. Limitation:

It is suggest that provide the limitation of your work.

Overall this study is a good paper with useful results but there are many flaws specially in methods and discussion. If the authors of the paper disagree with the comments/suggestions, then provide suitable justifications.
